# Phosphorylation by mTORC1 stablizes Skp2 and regulates its oncogenic function in gastric cancer

**DOI:** 10.1186/s12943-017-0649-0

**Published:** 2017-04-26

**Authors:** Qirong Geng, Jianjun Liu, Zhaohui Gong, Shangxiang Chen, Shuai Chen, Xiaoxing Li, Yue Lu, Xiaofeng Zhu, Hui-kuan Lin, Dazhi Xu

**Affiliations:** 1 State Key Laboratory of Oncology in South China, Collaborative Innovation Center for Cancer Medicine, Guangzhou, China; 2 Department of Hematology Oncology, Sun Yat-sen University Cancer Center, Guangzhou, China; 3 Department of Gastric Surgery, Sun Yat-sen University Cancer Center, 651# East Dongfeng road, Guangzhou, 510060 Guangdong Province China; 4 Institute of Biochemistry and Molecular Biology, Ningbo University School of Medicine, Ningbo, Zhejiang, 315211 China; 5 Department of Molecular and Cellular Oncology, M.D. Anderson Cancer Center, The University of Texas, 1515 Holcombe Boulevard, Houston, TX 77030 USA

**Keywords:** mTORC1, Phosphorylation, Skp2, Gastric cancer

## Abstract

**Background:**

Both mTOR and Skp2 play critical roles in gastric cancer (GC) tumorigenesis. However, potential mechanisms for the association between these two proteins remains unidentified.

**Methods:**

The regulatory role for mTORC1 in Skp2 stability was tested using ubiquitination assay. The functions of p-Skp2 (phosphorylation of Skp2) were studied in vitro and in vivo. Expression of p-Skp2 and p-mTOR (phosphorylation of mTOR) were shown in GC lines and in 169 human primary GC tissues.

**Results:**

mTORC1 can directly interact with Skp2 and phosphorylated Skp2 at Ser64, which sequentially protect Skp2 from ubiquitination and degradation. Furthermore, the phospho-deficient p-Skp2 (S64) mutant significantly suppresses GC cell proliferation and tumorigenesis. The expression of p-Skp2 was associated with p-mTOR in GC cell lines and tissues. Interestingly, the combination of p-Skp2 and p-mTOR was a better predictor of survival than either factor alone.

**Conclusion:**

The mTORC1 function to regulate Skp2 by Ser64 phosphorylation may represent an oncogenic event in GC tumorigenesis. Moreover, our study also indicates that Skp2 Ser64 expression is a potential indicator in the treatment of GC patients using mTORC1 inhibitor.

**Electronic supplementary material:**

The online version of this article (doi:10.1186/s12943-017-0649-0) contains supplementary material, which is available to authorized users.

## Background

Gastric cancer (GC) is still an important public health problem, with the third leading cause of cancer-related mortality worldwide [1]. The infection of Helicobacter pylori and Epstein-Barr (EB) virus is thought to be associated with carcinogenesis of gastric cancer [2–6].

Although D2 resection is the standard treatment for gastric cancer, most of GC patients are diagnosed in a non-curable stage, especially in China. The understanding of the molecular mechanisms is helpful to develop effective treatment strategies. It has been known that several signaling pathways play critical roles in the development of gastric cancer. For example, there is an increasing interest in studying the role of the mammalian target of rapamycin (mTOR) in gastric cancer. We have also revealed that mTOR is frequently activated in GC and phosphorylated mTOR is associated with poor prognosis [7].

mTOR can form one of two complexes: mTOR complex 1 (mTORC1) and mTOR complex 2 (mTORC2). Activated mTORC1 phosphorylates two effector molecules, 4E-binding protein 1 (4EBP1) and S6 kinase 1 (S6K1), to stimulate protein synthesis supporting cell growth and cell metabolism and angiogenesis [8–10].

As an oncoprotein, S-phase kinase associated protein 2 (Skp2) has also been reported activated in many types of cancers, included approximately 50% patients with GC [11–13]. It is the key part of Skp-Cullin-F-box (SCF) complex and participates in cell proliferation, metabolism and tumorigenesis by promoting ubiquitination of p27 and p21 [14–18]. Previously, we identified that macroh2A is a new Skp2 SCF substrate, whose ubiquitination by Skp2 inhibits the proliferation, colony formation and migration in breast cancer cells [19]. However, the upstream regulation of Skp2 remains unclear [20].

Recently, Jin et al. have found that Skp2 is a negative feedback regulator of amino-acid dependent mTORC1 signaling and activation of mTORC1 signaling can recruit Skp2 to RagA [21]. This result indicates mTORC1 activity can influence Skp2 activity and it is possible that Skp2 phosphorylation mediated by mTORC1 [22].

Here we evaluated the mechanism by which mTORC1 controls Skp2 stability at the phosphorylation level. Our findings may help to understand the role of mTOR-dependent networks and their relationship to compensatory pathways in GC tumorigenesis.

## Methods

### Cell lines, stable cell lines and plasmids

Human GC cell lines (BGC 823, MKN 45, SGC 7901 and MGC 803) and 293 T cell line were purchased from American Type Cell Culture (Manassas, VA). All cell lines were cultured in high-glucose RPIM-1640 or DMEM supplemented with 10% fetal bovine serum (Gibco) in a moist chamber with 5% CO_2_ at 37 °C.

Two cell lines (BGC 823 and MKN 45) were selected to generate stable cell lines in our research. The retroviral packaging system was purchased from Clontech. According to the manufacture’s introduction, the recombinant retroviruses expressing the vector pcDNA 3.1 and Skp2-wild-type (WT) or Skp2-S64A were generated. These retroviruses were infected to BGC 823 and MKN 45 and then were selected with 750ug/ml of G148 (Calbiochem). Mutation Skp2 was constructed by the Quik Change Site-Directed Mutagenesis Kit (Stratagene), and then verified by DNA sequencing. Follow the manufacture’s introduction, the plasmids containing pcDNA3.1-myc-mTOR, pcDNA3.1-FLAG-SKP2-wt and pcDNA3.1-FLAG-SKP2-S64A were constructed.

### Transfections, cell synchronization and cycloheximide chase assay

Cell lines were transfected with variously plasmids with lipofectamine 2000 (Life technologies) according to the manufacture’s introductions. Generally, cells seeds at 2.0 × 10^5^ cells per well in a 6-well culture dish or at 1.0 × 10^6^ cells per 10-cm dish were transfected with various plasmids. For the synchronization experiment, 293 T cells were treated with 2 mM thymidine for 12 h, and washed and released into fresh medium tissue culture dishs. As indicated, transfection were performed during the last 4 h before the second thymidine treatment. After the double-thymidine block, the 293 T cells were re-plated and treated with cycloheximide (100ug/ml) for 0 h to 5 h. Then, the Skp2, mTOR and GAPDH were detected by immunoblotting.

### Antibodies and reagents

Antibodies against Skp2, mTOR, phosphorylate-mTOR-ser2448, S6K, phosphorylate-S6K-ser389, 4EBP1, phosphorylate-4EBP1-ser65 were purchased from Santa Cruz Biotechnology Inc. Antibodies against FLAG, myc, His were purchased from Sigma-Aldrich. Phosphorylate-Skp2-ser64 was generated by Zoonbio Biotechnology Co.,Ltd. The mTOR inhibitor rapamycin were obtained from Sigma.

### RNAi treatment

All siRNAs were produced by GenePharma. According to the manufacturer’s protocol, 293 T cells were transfected with siRNA oligonucleotides using the Lipofectamine RNAiMAX transfection reagent (Invitrogen) for 48 h.

The siRNAs for mTOR are as follows:

mTOR no.1 sense:5’-GCAUCCAGCAGGAUAUCAATT-3’; antisense:5’-UUGAUAUCCUGCUGGAUGCTT-3’

mTOR no.2 sense:5’- GCUGUCAGCCUGUCAGAAUTT -3’; antisense:5’- AUUCUGACAGGCUGACAGCTT-3’

### Immunoblotting and immunoprecipitation

Whole-cell extracts were lysed by lysis buffer (1 × Cell Lysis Buffer (Cell Signaling Technology) adding 1 mM phenylmethylsulphonyl fluoride (PMSF) immediately before use). Protein suspension liquid were boiled after addition 6 × SDS sample buffer for 5–10 min at 100 °C. In immunoprecipitation experiment, whole-cell were lysed by E1A lysis buffer (50 mM HEPES (pH 7.5), 250 mM NaCl, 5 mM EDTA, 0.1% NP-40, protease inhibitor cocktail (Roche)). Immunoprecipitation was carried out either by incubating appropriate antibody with cell lysate for 2–3 h, followed by incubating Protein-A/G beads overnight (Roche) or by incubating FLAG beads at 4 °C with lysate overnight. Beeds were washed with ice-cold PBS three times, resuspended in SDS loading buffer. The protein samples were resolved by 10% SDS-PAGE and transfered to membrane (Sigma-aldrich). Then, the blots were identified by various antibodies.

### In vivo ubiquitination assay

In vivo ubiquitylation assays were performed as described^19^. In brief, 293 T cells were transfected with the indicated plasmids for 24 h, and were treated with 20 μM LY, or 100 nM Wortmannin together with 20 μM MG132 for 6 h prior to harvesting. The cell extracts were then incubated with nickel beads for 3 h, washed, and subjected to the Western blot analysis.

### Cell growth and in vivo tumorigenesis assay

For cell growth assay, 5 × 10^3^BGC823 stable cells with pcDNA, pcDNA-Skp2, or pcDNA-Skp2 S64A and pcDNA-Skp2 S64D were seeded in 12-wells in triplicate, harvested, and stained with trypan blue at different days. Numbers of viable cells were directly counted under the microscope. The individual clone was picked and verified by Western blot analysis.

For in vivo tumorigenesis assays, BGC823 stable cells (1 × 10^6^) mixed with matrigel (1:1) were injected subcutaneously into the left flank of 6-week-old nude mice. Tumor size was measured weekly with a caliper, and the tumor volume was determined using the standard formula: L × W2 × 0.52, where W is the shortest diameter and L is the longest diameter.

### Patients and specimens

GC samples and the adjacent nontumor samples were obtained from 169 GC patients who underwent radical resection for histologically confirmed gastric cancer from Sun Yat-sen University Cancer Center between January 2000 and December 2004. All patients had follow-up after surgery at 6 – 12 month intervals; the final date of follow-up was on Jan 1, 2015. The primary end point of our study was Overall survival (OS). Disease-free survival (DFS) was the time between the time of surgery and relapse or tumor-related death. Overall survival was defined as the period between the time of surgery and death.

Immunohistochemistry was done by means of a standard of protocol. Expression of p-mTOR and p-Skp2 were examined by at least two investigators who were blinded to clinical data. Immunostaining was classified based on staining intensity and percentage of p-mTOR-positive and p-Skp2-positive tumor cells. Staining intensity was determined as 0 (absent), 1 (weak), and 2 (strong). Expression levels were semi-quantified using an immunohistochemistry score (range, 0–200) calculated by the percentage of positive tumor cells. Patients with an immunochemistry score of >20 as p-m-TOR and p-Skp2 positive or high expression and those with a score of ≤20 were considered as p-mTOR and p-Skp2 negative or low expression.

## Results

### Skp2 protein level is regulated by the mTORC1 in gastric cancer cells

**Fig. 1:**
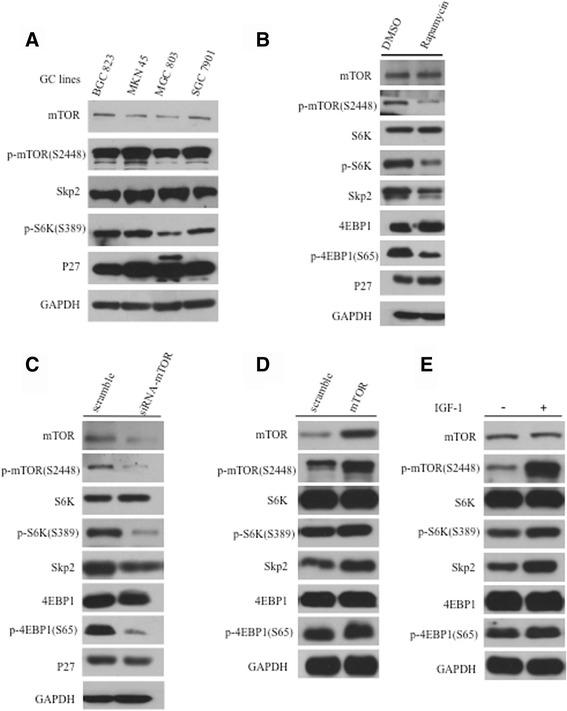
Skp2 is regulated by the mTORC1 in gastric cancer cells. **a** The expression of Skp2 was associated with phosphorylate mTOR in different gastric cancer cell lines. (**b**, **c**) mTORC1 pathway prevented by rapamycin (**b**) and siRNA (**c**) in BGC 823 cells downregulated the expression of Skp2. (**d**, **e**) The enhancement of mTOR activity by overexpression mTOR (**d**) or IGF-1 (**e**) in BGC 823 cells leads to upregulation of Skp2 expression. The 293 T cell were treated IGF-1 at various time points after starvation 12 h

To investigate whether mTOR signaling regulate Skp2 expression in gastric cancer, we detected the expression of mTOR and Skp2 in cell lines. Firstly, as shown in Fig. 1a, there was a positive correlation between mTORC1 activity and Skp2 expression in a panel of GC cell lines. Secondly, we treated BGC 823 (Fig. 1b) and MKN45 cells (Additional file 1: Figure S1A) with the mTOR inhibitor rapamycin, and observed decreased Skp2 protein levels concomitant with the inhibition of mTOR activity. Similar results were also obtained by knockdown mTOR in both BGC 823 (Fig. 1c) and MKN 45 cells (Additional file 1: Figure S1B). Furthermore, the enhancement of mTORC1 activity by overexpression mTOR (Fig. 1d) or insulin-like growth factor (IGF)-1 (Fig. 1e) in BGC 823 cells leads to upregulation of Skp2 expression, suggesting that elevated mTOR activity upregulaties Skp2 in these two cell lines. These findings indicate that Skp2 is a candidate substrate of mTORC1.

### mTORC1 interacts with Skp2

**Fig. 2:**
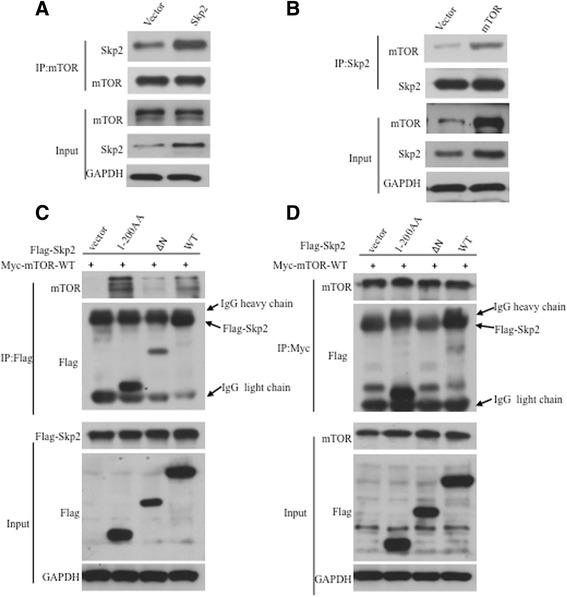
Skp2 interacts with mTOR. **a** Endogenous mTOR interacts with exogenous Skp2. 293 T cells were transfected with the indicated plasmids and harvested for immunoprecipitation experiments. **b** Endogenous Skp2 interacts with exogenous mTOR. 293 T cells were transfected with the indicated plasmids and harvested for coimmunoprecipitation experiments. (**c**, **d**) 293 T cells were contransfected with indicated plasmids and harvested for immunoprecipitation assay, indicating N-terminal of Skp2 (amino acids 1–200) were required for the interaction between mTORC1 and Skp2

Next, the interaction between mTORC1 and Skp2 was tested. As seen in Fig. 2a, b, using co-immunoprecipitation, we found that mTORC1 could interact with Skp2 in HEK293T cells overexpressing Flag-tagged Skp2 or Myc-tagged mTORC1. Moreover, endogenous mTORC1 and Skp2 was also interacted in BGC 823 cells (Additional file 1: Figure S1C). To narrow down the sequences responsible for the interaction between mTORC1 and Skp2, we generated a series of deletions of Skp2 and performed co-immunoprecipitation. Clearly, we demonstrated that N-terminal of Skp2 (amino acids 1–200) were required for the interaction between mTORC1 and Skp2 (Fig. 1c, d).

### mTORC1 phosphorylates Skp2 at Ser64

**Fig. 3:**
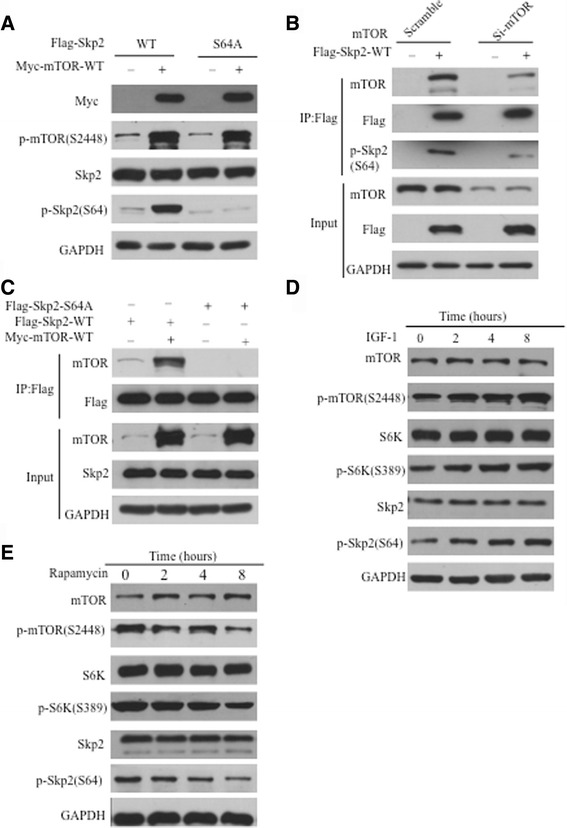
mTORC1 phosphorylate Skp2 on Ser64. **a** 293 T cells were contransfected with Flag-Skp2-S64A and Myc-mTOR-WT for 48 h. The cell lysates were analyzed by Western blot. **b**, **c** 293 T cells were transfected with the indicated plasmids and harvested for co-immunoprecipitation experiments. **d** The 293 T cell were treated IGF-1 at various time points after starvation 12 h. The cell lysates were analyzed by Western blot. **e** The 293 T cell were treated with rapamycin at various time points. The cell lysates were analyzed by Western blot

The GPS 2.0, a tool to predict kinase-specific phosphorylation sites, showed that there are additional putative mTORC1 phosphorylation sites present in human Skp2, including Ser64, Ser96, Ser168 and Th225. Next, we performed mass spectrometry analysis to validate these sites [23]. An 80KDa mass shift was observed clearly on Ser64, suggesting that Ser64 is the main site for mTORC1-mediated Skp2 phosphorylation (Additional file 1: Figure S2A). The motif surrounding Ser64 is highly conserved in Skp2 in all mammals, including humans and mice (Additional file 1: Figure S3A). Furthermore, we generated a polyclonal phospho-Skp2-S64 specific antibody. Using this antibody, myc-active-mTORC1 can phosphorylate Skp2, but not Skp2 S64A (Fig. 3a). As shown in Fig. 3b, p-Skp2-S64 was clearly detected in the cells transfected with WT-mTOR, but phosphorylation signal decrease in knock-down mTOR cells. These results suggested that phosphorylation on Ser64 of Skp2 was dependent upon mTORC1 kinase activity. It was further confirmed by the observations that the interaction between Skp2 and mTORC1 was abolished by the Skp2 S64A (Fig. 3c). The phenomenon also suggested that mTORC1 binds to Skp2 in a phosphorylation-dependent manner and the Ser64 motif of Skp2 is important for binding to mTORC1 as well as regulating Skp2.

When IGF-1 activate mTORC1 pathway in BGC-823 gastric cancer cells, endogenous Skp2 was phosphorylated at Ser64 (Fig. 3d). Importantly, rapamycin can dramatically reduced its phosphorylation (Fig. 3e). In vitro mTOR kinase assay, we also demonstrated that mTORC1 phosphorylated WT Skp2, but not the S64A mutant (Additional file 1: Figure S3B). Collectively, these results showed that mTORC1 directly phosphorylates Skp2 at S64 both in vitro and in vivo.

### Phosphorylation of Skp2 at Ser64 by mTORC1 affects Skp2 protein stability

**Fig. 4:**
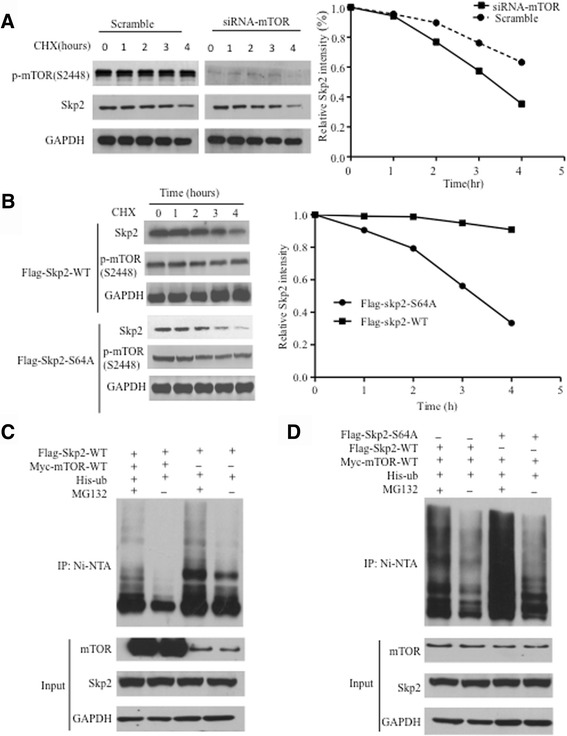
mTORC1 stabilizes Skp2 via ubiquitin/proteasome pathway. **a** 293 T cells transfected with Control or mTOR-siRNA were incubated with 20 μg/ml of CHX for the indicated times. Cell lysates were then analyzed by Western blot (left panel), and the densities of the Skp2 protein bands at each time points were normalized to GAPDH (right panel), indicating Skp2 degrades much faster in mTOR-depleted cells than control cells. **b** 293 T cells stably expressing Skp2-WT or the S64A mutant were incubated with 20 μg/ml of CHX for the indicated times. Cell lysates were then analyzed, indicating Skp2 displayed decreased half-life in cells expressing Skp2-S64A compared to those expressing Skp2-WT (**c**, **d**) 293 T cells were transfected with the indicated plasmids, followed by immunoprecipitation with nickel-agarose beads and then analyzed by Western blot. The ubiquitinated Skp2 decreased in mTOR overexpression cells, indicating that the degradation of Skp2 prevented by mTORC1 is ubiquitination dependent (**c**). In the presence of Skp2-S64A, Skp2 was degrades faster than Skp2-WT (**d**)

Given that depleting endogenous mTORC1 in BGC823 and MKN45 cells led to decreased expression of Skp2 (Fig. 1b, c and Additional file 1: Figure S1A and B), we surmised that mTORC1 might affect Skp2 protein stability. Indeed, when we treated the cells with protein synthesis inhibitor cycloheximide (CHX), Skp2 degrades much faster in mTOR-depleted cells than control cells (Fig. 4a). In support of a regulatory role for mTORC1 in Skp2 stability, Skp2 displayed decreased half-life in cells expressing S64A-Skp2 compared to those expressing WT-Skp2 (Fig. 4b). We next performed ubiquitination assay and found that the ubiquitinated Skp2 decreased in mTOR overexpression cells, indicating that the degradation of Skp2 prevented by mTORC1 is ubiquitination dependent (Fig. 4c). Furthermore, in the presence of mTORC1 overexpression and MG132 (a specific proteasome inhibitor), Skp2 was more heavily ubiquitinated (Fig. 4c, d). Notably, ubiquitination of Skp2 was increased in cells expressing S64A-Skp2 than expressing WT-Skp2 (Fig. 4d). Taken together, these data strongly demonstrated that mTORC1 phosphorylates and stablizes Skp2 through the ubiquitinproteasome system.

### mTORC1-mediated phosphorylation of Ser64 is critical for proto-oncogenic functions of Skp2 in gastric cancer tumorigenesis

**Fig. 5:**
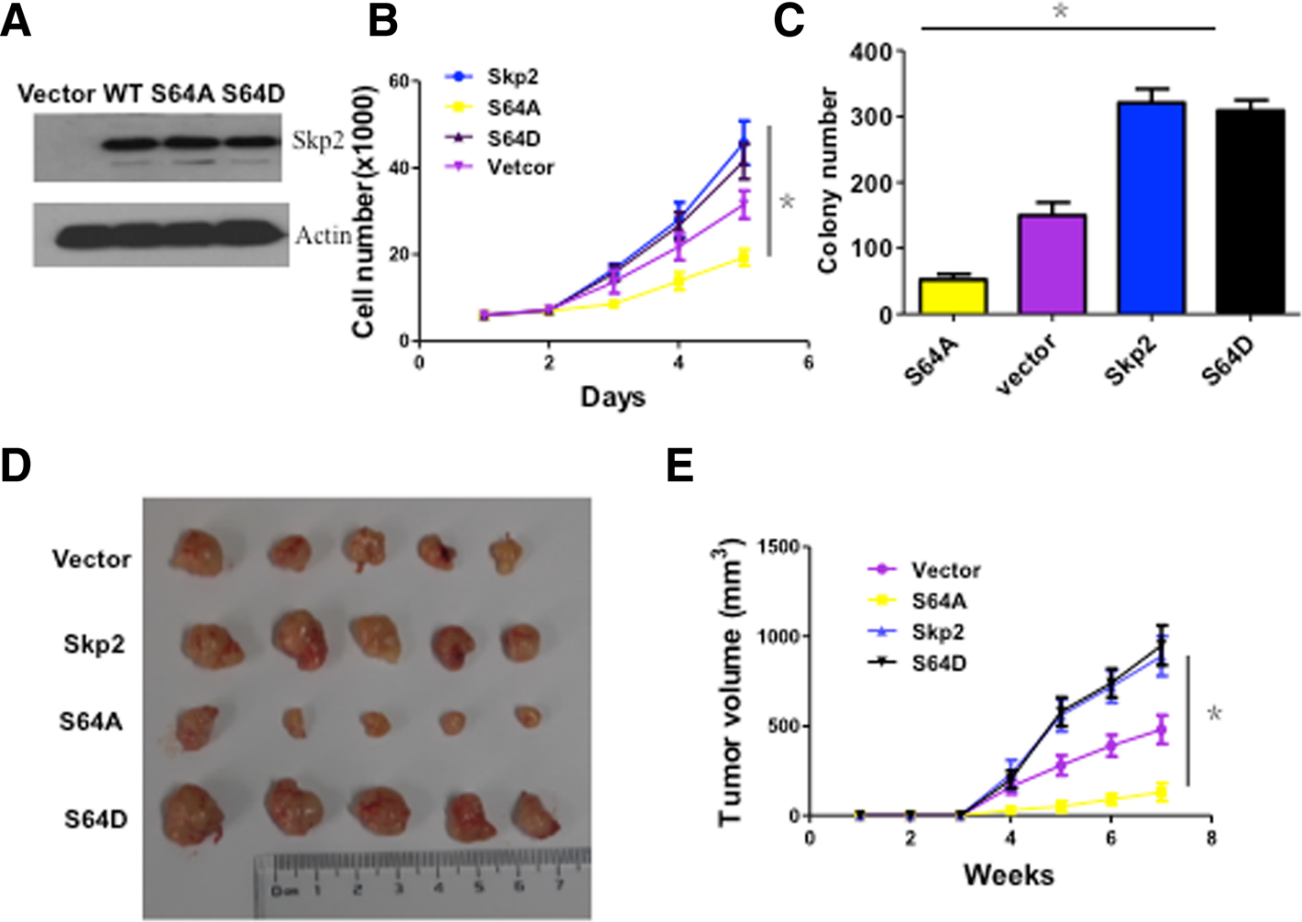
Skp2 S64 phosphorylated by mTOR is critical for gastric cancer cell proliferation. **a** BGC823 cells were transfected with the indicated plasmids for 48 h and harvested for Western blot analysis. (**b**, **c**) BGC823 cells with stable overexpression of Vector,WT-Skp2,Skp2 S64A or Skp2 S64D were plated for cell growth analysis (**b**) and soft agar transformation assay (**c**). Results are presented from a representative experiment. *p < 0.05, using Student’s *t*-test, *n* = 3. **d** BGC823 cells stably transfected with Vector,WT-Skp2,Skp2 S64A or Skp2 S64D were injected into nude mice (*n* = 5 for each group) for tumorigenesis. **e** Pictures were taken 7 weeks after the injection. *p < 0.05 using Student’s *t*-test

To assess whether Skp2 phosphorylation by mTORC1 would affect its biological functions, we generated BGC823 cells with stable overexpression of WT-Skp2 or S64A-Skp2 for cell proliferation and oncogenic transformation (Fig. 5a). As shown in Fig. 5b, overexpression of Skp2 WT and Skp2 S64D markedly accelerated cell growth, whereas the cells stably transfected with the Skp2 S64A displayed less rapid cell proliferation. Next, in a colony formation assay, we showed that the cells expressing Skp2 WT and Skp2 S64D formed a higher number of colonies than the Vector and Skp2 S64A transfected cells (Fig. 5c). Similar results can be found in MKN45 cells (Additional file 1: Figure S4A and B). Additionaly, in the transwell assay experiment included Skp2 S64A and Skp2 siRNA in BGC823 cells (Additional file 1: Figure S4C), the cells with Skp2-WT has the most powerful invasion ability, followed by cells with Vector, Skp2-S64A and Skp2-siRNA. However, cells with Skp2-S64A and Skp2-siRNA showed no significant difference in invasion ability.

Moreover, in animal model, when we injected BGC823 cells with empty vector, Skp2 WT, or Skp2 mutants subcutaneously into nude mice, Skp2 WT and Skp2 S64D profoundly promoted tumorigenesis when compared to the control, whereas Skp2 S64A completely lost this activity (Fig. 5d, e).

These findings demonstrate that in Skp2 S64A tumor cells in which S64 is constitutively inactivated, the tumor oncogenic activity of Skp2 is abrogated in vitro and in vivo by preventing the phosphorylation of Skp2 by mTORC1.

### Clinical Relevance of p-Skp2 (S64) in human gastric cancer

**Fig. 6:**
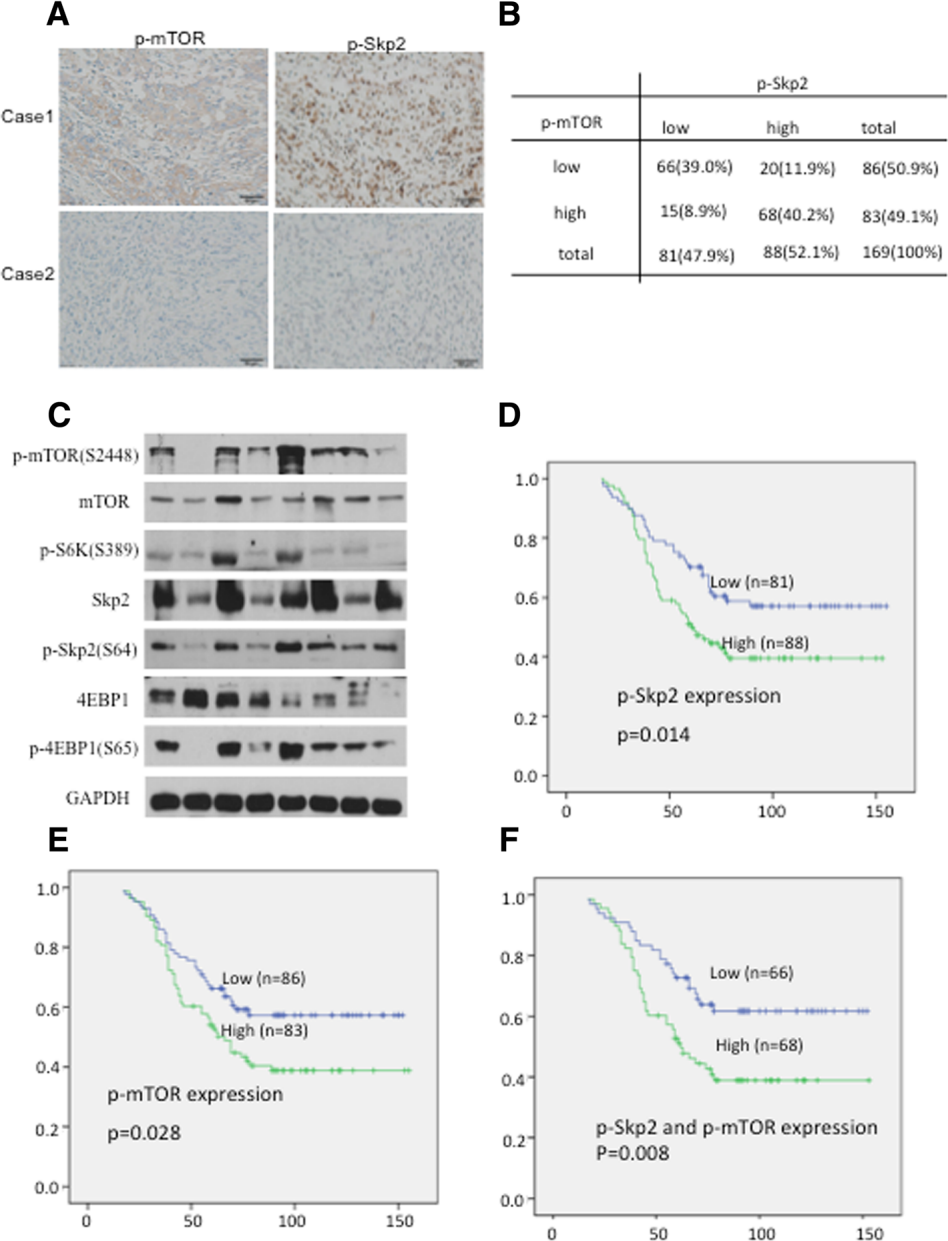
Clinical relevance of p-Skp2 (S64) in human gastric cancers. **a** Representative IHC staining of p-Skp2 (S64) and p-mTOR (S2448) expression in GC; scar bar, 50um. **b** The correlation between p-mTOR and p-Skp2 expression was analyzed by Spearman rank correlation test (R=0.59; *p* < 0.01). **c** Representative GC tissues were extracted and analyzed by western bolt. (**d**-**f**) The Kaplan-Meier overall survival curves indicated that p-Skp2 (S64) and p-mTOR (S2448), alone (**d**, **e**) and together (**f**), are associated with a reduction of overall survival in gastric cancer patients

To further determine whether the aforementioned findings could be supported in human GCs, we analyzed the expression of p-Skp2 (S64) and p-mTOR (S2448) in primary GC specimens. Of 169 cases with immunohistochemical staining (Fig. 6a), 88 cases showed p-Skp2 staining in carcinoma cells. Notably, p-Skp2 was detected in 68 (82%) of the 83 specimens with high p-mTOR expression, but in only 20 (23%) of the 86 specimens with low p-mTOR expression. Spearman rank correlation test showed that p-Skp2 expression was strongly associated with p-mTOR expression (R=0.59; *p* < 0.01) (Fig. 6b). We also found that p-Skp2 expression strongly correlates with lymph node (LN) metastasis in GCs (Table 1, *p* = 0.03) and pTNM stage (Table 1, *p* = 0.02).

**Table 1:** Clinicopathologic correlation for p-Skp2 expression in GC patients

Factors	All patients	No. of patients		*p*
	*N* = 169	p-Skp2 negative	p-Skp2 positive	
Age (y)				0.14
<60	84	45	39	
≥60	85	36	49	
Sex				0.39
Male	121	55	66	
Female	48	26	22	
Site				0.04
Upper	72	27	45	
Middle	25	12	13	
lower	72	42	30	
Tumor size				0.68
≤4 cm	62	31	31	
>4 cm	107	50	57	
Pathologic T classification				0.16
T1	17	12	5	
T2	32	12	20	
T3	41	18	23	
T4	79	39	40	
Pathologic N status				0.03
N negative	41	26	15	
N positive	128	55	73	
Pathologic stage (pTNM)				0.02
I	23	17	6	
II	45	21	24	
III	101	43	58	
p-mTOR expression				<0.0005
negative	86	66	20	
positive	83	15	68	

Additionally, we performed quantitative RT-PCR (qRT-PCR), immunoblotting, and immunohistochemistry (IHC) assays using 20 freshly prepared GC tissues matched with adjacent nontumor tissues (NTs). We showed that p-Skp2 (S64) was overexpressed compared with adjacent NTs (Additional file 1: Figure S5A–C), further implying that phosphorylation of Skp2 at Ser64 by mTORC1 may represent an oncogenic event in GC. In agreement with previous results, the tumor immunohistochemical staining data indicates that the mTORC1 pathway regulates Skp2 expression through phosphorylation of Skp2 (Fig. 6c).

Furthermore, the Kaplan-Meier overall survival curves showed that high p-Skp2 (S64) and p-mTOR (S2448) expressions were associated with poor survival. Interestingly, the combination of p-Skp2 and p-mTOR was a much better predictor of survival than either factor alone (*p* < 0.01 versus *p* < 0.05) (Fig. 6d–f).

## Discussion

In this study, we showed that Skp2 could be regulated by ubiquitin-mediated degradation, which promoted by mTORC1, a central regulatory kinase in key cellular process. Specifically, we found that mTORC1 directly interacts with Skp2 in a phosphatase-independent manner. Conversely, both siRNA and mTOR inhibitor rapamycin disrupts the activity of Skp2. Taken together, these results indicate that the phosphorylation of Skp2 at Ser64 is mainly mediated by mTORC1.

Previously, some kinases have been shown to phosphorylate Skp2 by controlling its stability. For example, phosphorylation of Skp2 by CDK2 at Ser64 and Ser72 can protect it from degradation [24]. Akt1 and Pim-1 also appears capable of phosphorylating Skp2 at Ser72 [25, 26]. However, Unlike Akt1, Pim-1 kinase could not regulate Skp2 subcellular localization by phosphorylation [26]. It suggests that the regulating role of phosphorylation of Skp2 is decided by the different kinases [27]. Moreover, the critical role of Skp2 phosphorylation in cancer cell has not yet been explored.

Indeed, mTORC1 has emerged as an important player in the regulation of Skp2. For example, Shapira et al. revealed that the mTOR inhibitor rapamycin significantly decreased Skp2 levels and enhanced the degradation of Skp2. p27 levels were also up-regulated in rapamycin-sensitive cells [28] Therefore, clarifying the mechanism how mTORC1 regulates Skp2 has important implications for cancer therapy [28, 29].

Currently, our results demonstrated that phosphorylation on Ser64 by mTORC1 is required for the stabilization of Skp2 levels in GC. In GC cells, we showed that the mTOR siRNA and Skp2 S64A mutation inhibited cell transformation and proliferation than WT in vitro and in vivo. Notably, in a panel of GC cell lines, there is a positive correlation between the elevated expression of mTOR and p-Skp2 (Ser64). These results implicate mTORC1 as an upstream regulatory factor for the Skp2 pathway at the posttranslational level and overexpression of mTORC1 may contribute to the activation of Skp2 oncogenic function in GC.

Recent studies have revealed that p-mTOR expression is upregulated in GCs [30, 31]. However, the expression of p-Skp2 (Ser64) and their correlation in GCs remain to be investigated. In the current study, we firstly showed that p-Skp2 overexpression in advanced GCs and can predict poor survival outcome for patients. Importantly, 83 out of 169 cancer tissues examined contained high expression of p-mTOR, whereas 68 out of the 88 tissues containing active Skp2, supporting the conclusion that activation of mTOR signaling is involved in the overproduction of Skp2. On the other hand, p-Skp2 (S64) was undetected in 81 of the 86 specimens with low p-mTOR expression, this suggests that decreased p-mTOR can predict the inactivation of Skp2 by reducing phosphorylation of Skp2. Furthermore, our study demonstrated that the combined expression of p-mTOR and p-Skp2 is much better to predict the survival of GC patients than each of them. Collectively, the clinical data further strengthened the notion that phosphorylation of Skp2 by mTORC1 protect Skp2 from degradation and promotes Skp2 activation and is associated with the prognosis of GC patients.

Our results have important clinical implications in the treatment of GC for several reasons. First, it is helpful to identify which subgroup of GCs may respond the most to mTOR inhibitor. To date, some clinical trials involving rapalogues have been performed in GC patients, with negative results [32]. For example, in 2013, a randomized clinical trial showed that mTOR inhibitor everolimus failed to improve the survival of GC patients [33]. The main reason may be that the patients particularly dependent on mTOR signaling were not identified [34]. Indeed, given that cells that express elevated Skp2 Ser64 have better sensitivity to rapamycin, Skp2 Ser64 expression would be a potential biomarkers in the treatment of GC patients using mTOR inhibitor. Secondly, our data also provide a new view on cancer therapy by preventing the phosphorylation of Skp2. Recently, Chan CH et al. have reported Skp2 inhibitor can exhibits potent antitumor effect in many cancers by targeting cancer stem cell [35]. It will be interesting to explore the effect of Skp2 S64 phosphorylation on GC stem cell. Additionally, combination target-therapy have emerged and showed promising anticancer activities [36]. Since constitutive activation of the mTORC1-Skp2 pathway frequently occurs in GCs and combined their expression has worse prognosis, it is very likely that the intervention by combining Skp2 inhibitors and mTOR inhibitors may be a much better approach for GC therapy.

## Conclusions

In conclusion, the present study provides the first evidence of how Skp2 is regulated by mTORC1. mTORC1 may function to regulate Skp2 by serving as a priming kinase, which stablizes Skp2 and consequently leads to its oncogenic effects on gastric cancer tumorigenesis. Importantly, our study indicates that p-Skp2 (Ser64) expression is a hopeful biomarker for the subset of GC patients that benefit from mTOR inhibitor. In addition, our data also suggest that combining Skp2 inhibitors and mTOR inhibitors might represent a potentical therapeutic strategy in the treatment of GC. Further studies are required to test this possibility.

## Additional file

Additional file 1: Figure S1. mTORC1 pathway prevented by rapamycin (A) and siRNA (B) in MKN45 cells downregulated the expression of Skp2. mTOR (Endogenous) interacts with Skp2 (Endogenous) in BCG823 cell line (C). **Figure S2.** Mass Spectrometry identified Ser64 phosphorylation of Skp2 by mTORC1. **Figure S3.** Sequence alignment of Skp2 with the Ser64 site recognized by mTORC1 in a variety of species (A). Flag-tagged Skp2 WT protein translated in vitro was incubated with mTOR WT or mTOR KD for 2 h. The samples were then analyzed by Western blot (B). **Figure S4.** MKN45 cells with stable overexpression of Vector,Skp2 WT,Skp2 S64A and Skp2 S64D were plated for cell growth analysis (A) and soft agar transformation assay (B). BGC823 cell lines with Vector, Skp2 WT, Skp2 S64A and Skp2-siRNA were plated for invasion analysis (C). Results are presented from a representative experiment. **p* < 0.05, using Student’s *t*-test, *n* = 3. **Figure S5.** Ten pairs of GC tissues and the non-tumor counterparts were extracted and exposed to qRT-PCR to determine mRNA levels (A), which were normalized to the internal GAPDH(B). Representative gastric cancer tumor tissues (T) and adjacent normal tissue (N) were extracted and analyzed by western bolt (C). Representative IHC staining of p-Skp2 (Ser64) in serial sections of human gastric cancer specimens. T, tumor; ANT, adjacent normal tissue; scale bar, 50um. (PDF 4776 kb)

## Abbreviations

4EBP1: 4E-binding protein 1
CHX: Cycloheximide
DFS: Disease free survival
EB: Epstein-Barr
GC: Gastric cancer
mTORC1: mTOR complex 1
mTORC2: mTOR complex 2
OS: Overall survival
p-mTOR: Phosphorylation of mTOR
p-Skp2: Phosphorylation of Skp2
S6K1: S6 kinase 1
Skp2: S-phase kinase associated protein 2
