# Supplementary material for: Phosphorylation by mTORC1 stablizes Skp2 and regulates its oncogenic function in gastric cancer
Source: Mol Cancer. 2017 Apr 26;16:83. doi: 10.1186/s12943-017-0649-0 (PMC5407005; doi:10.1186/s12943-017-0649-0)
Supplement: Additional file 1: Figure S1. — mTORC1 pathway prevented by rapamycin (A) and siRNA (B) in MKN45 cells downregulated the expression of Skp2. mTOR (Endogenous) interacts with Skp2 (Endogenous) in BCG823 cell line (C). Figure S2. Mass Spectrometry identified Ser64 phosphorylation of Skp2 by mTORC1. Figure S3. Sequence alignment of Skp2 with the Ser64 site recognized by mTORC1 in a variety of species (A). Flag-tagged Skp2 WT protein translated in vitro was incubated with mTOR WT or mTOR KD for 2 h. The samples were then analyzed by Western blot (B). Figure S4. MKN45 cells with stable overexpression of Vector,Skp2 WT,Skp2 S64A and Skp2 S64D were plated for cell growth analysis (A) and soft agar transformation assay (B). BGC823 cell lines with Vector, Skp2 WT, Skp2 S64A and Skp2-siRNA were plated for invasion analysis (C). Results are presented from a representative experiment. *p < 0.05, using Student’s t-test, n = 3. Figure S5. Ten pairs of GC tissues and the non-tumor counterparts were extracted and exposed to qRT-PCR to determine mRNA levels (A), which were normalized to the internal GAPDH(B). Representative gastric cancer tumor tissues (T) and adjacent normal tissue (N) were extracted and analyzed by western bolt (C). Representative IHC staining of p-Skp2 (Ser64) in serial sections of human gastric cancer specimens. T, tumor; ANT, adjacent normal tissue; scale bar, 50um. (PDF 4776 kb) [file 12943_2017_649_MOESM1_ESM.pdf]

Additional files

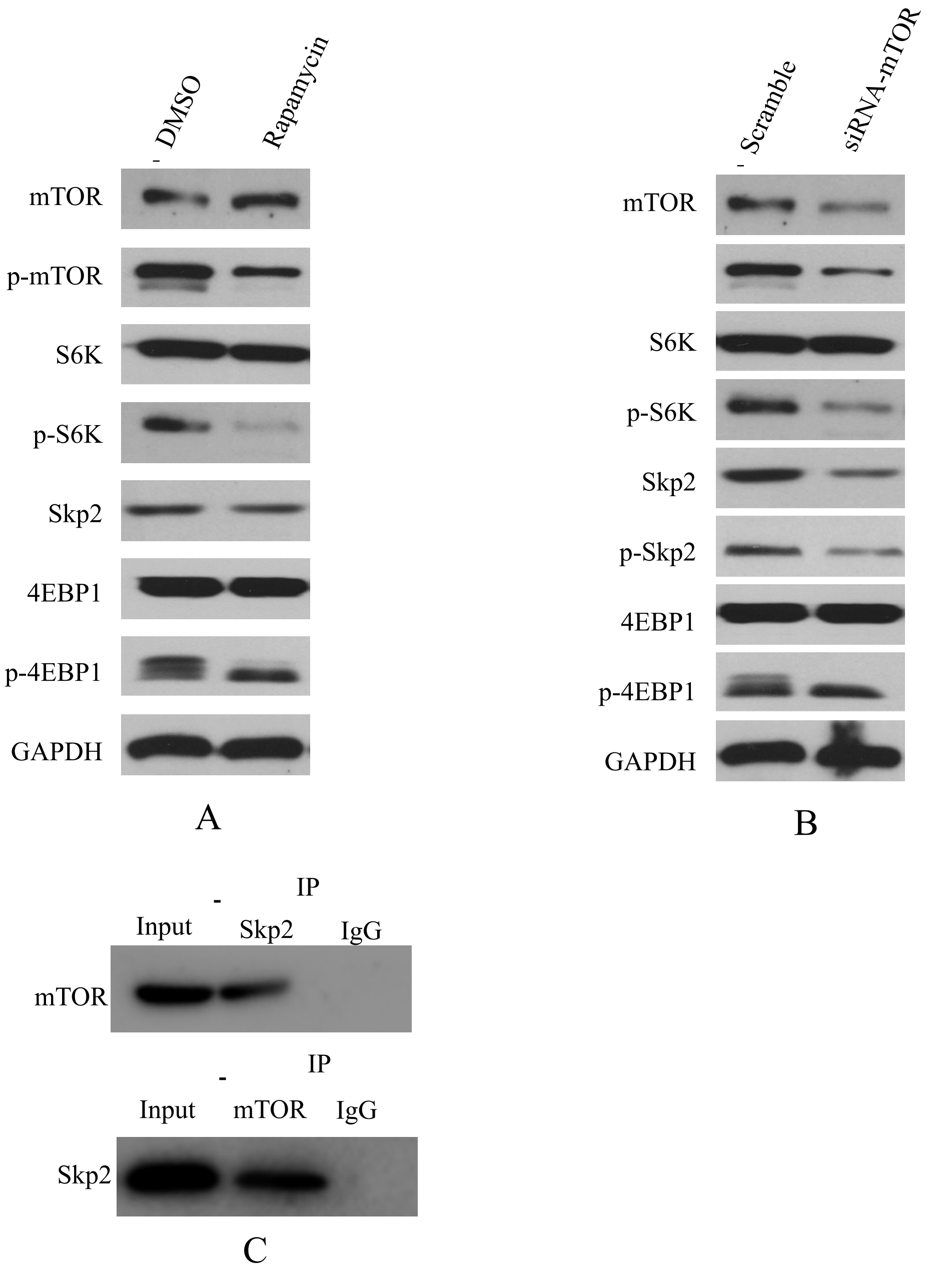

Figure S1. mTORC1 pathway prevented by rapamycin (A) and siRNA(B) in MKN45 cells downregulated the expression of Skp2. mTOR (Endogenous) interacts with Skp2 (Endogenous) in BCG823 cell line (C).

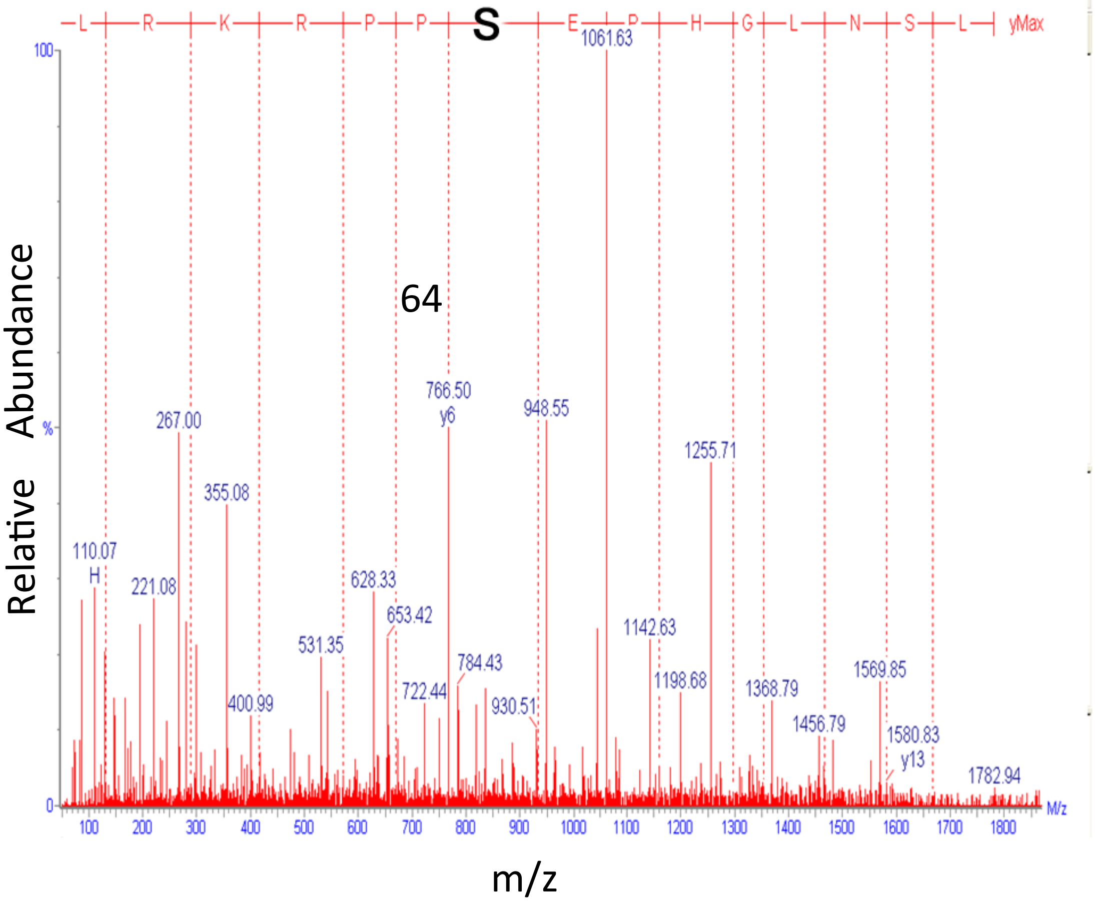

Figure S2. Mass Spectrometry identified Ser64 phosphorylation of Skp2 by mTORC1

	Ser-64
Skp2 consensus motif	
Homo sapiens	GHPE **S** PPRKRL
Rattus norvegicus	GHPQ **S** PPRKRL
Mus musculus	GHPQ **S** PPRKRV
Macaca mulatta	GHPE **S** PPRKRL
Canis familiaris	GHPQ **S** PPRKRL
Bos taurus	GHPQ **S** PPRKRL
Sus scrofa	GHPQ **S** PPRKRL
Spermophilus kateralis	GHPQ **S** PTRKRL
Pan troglodytes	GHPE **S** PPRKRL

A

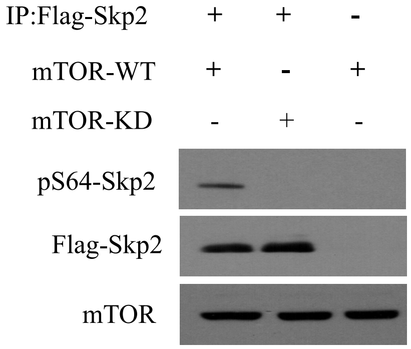

B

Figure S3. Sequence alignment of Skp2 with the Ser64 site recognized by mTORC1 in a variety of species (A). Flag-tagged Skp2 WT protein translated in vitro was incubated with mTOR WT or mTOR KD for 2 hours. The samples were then analyzed by Western blot (B).

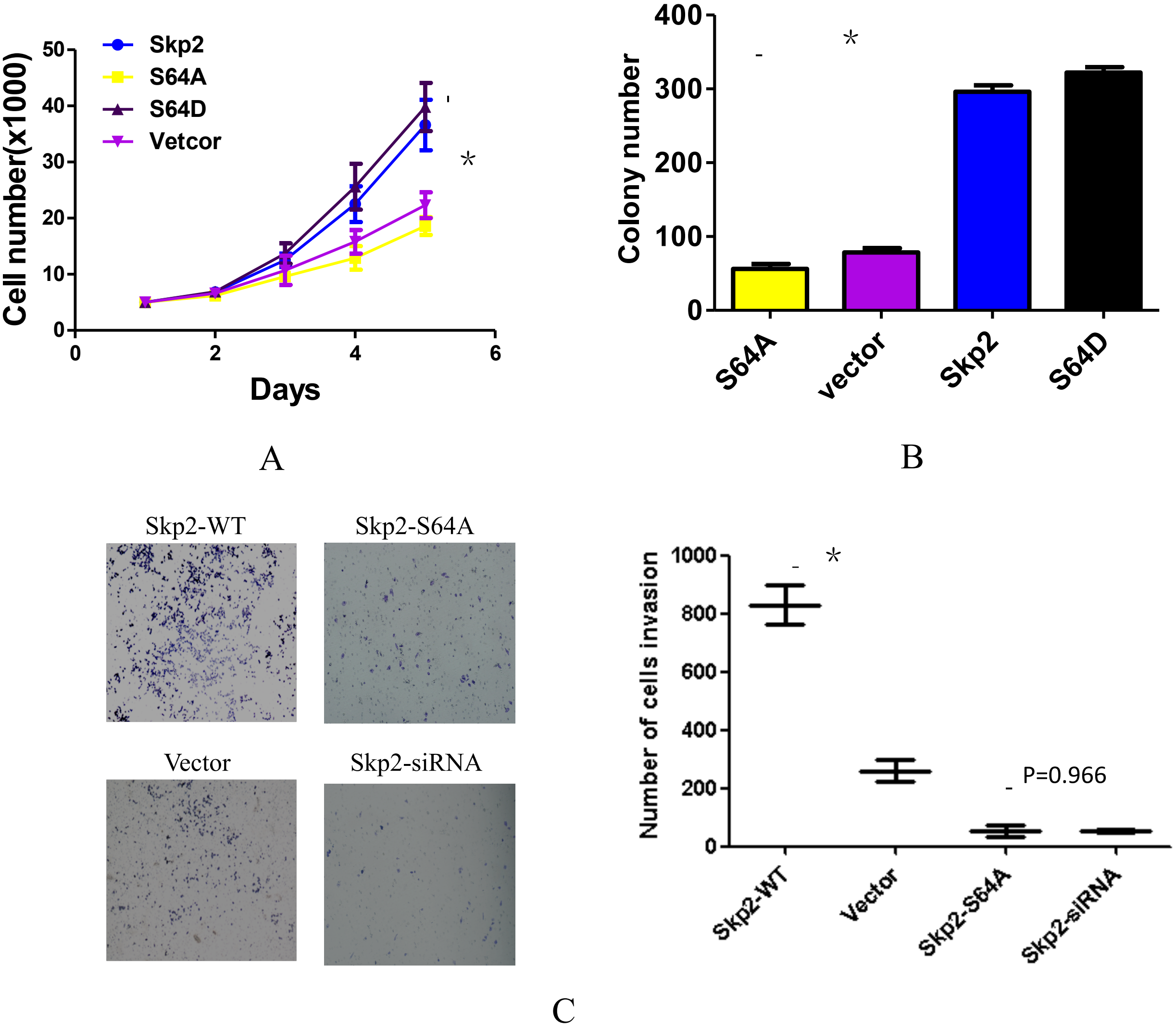

Figure S4. MKN45 cells with stable overexpression of Vector,Skp2 WT,Skp2 S64A and Skp2 S64D were plated for cell growth analysis(A) and soft agar transformation assay (B); BGC823 cell lines with Vector, Skp2 WT, Skp2 S64A and Skp2-siRNA were plated for Invasion sults are presented from a representative experiment.
*p<0.05, using Student's t-test, n=3.

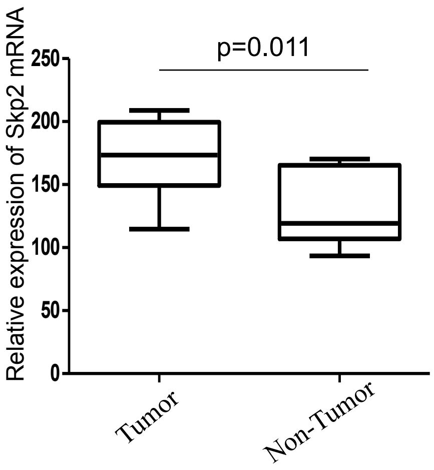

A

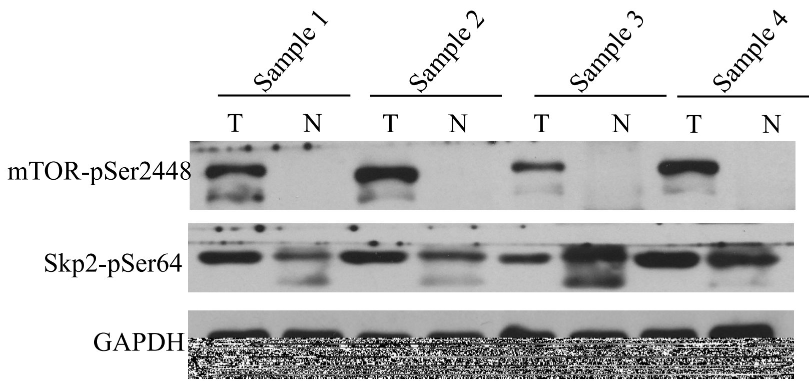

B

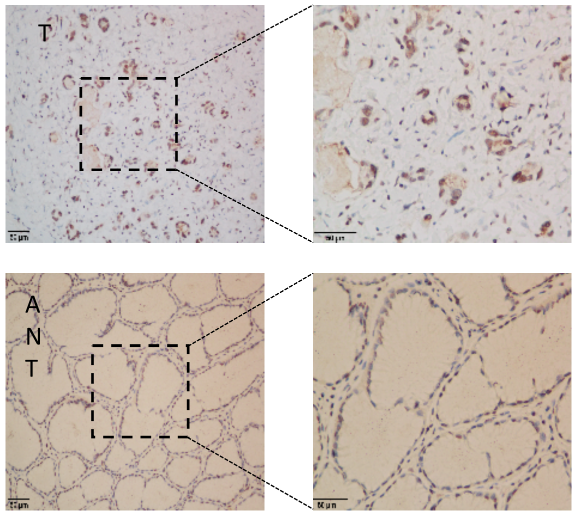

C

Figure S5. Ten pairs of GC Qssues and the non-tumor counterparts were extracted and exposed to qRT-PCR to determine mRNA levels(A), which were normalized to the internal GAPDH(B). Representative gastric cancer tumor tissues (T) and adjacent normal tissue (N) were extracted and analyzed by western bolt (C). Representative IHC staining of p-Skp2 (Ser64) in serial sections of human gastric cancer specimens. T, tumor; ANT, adjacent normal tissue; scale bar, 50um.
